# Therapeutic potential of seaweeds and their biofabricated nanoparticles in treating urolithiasis: A review

**DOI:** 10.1016/j.heliyon.2024.e41132

**Published:** 2024-12-11

**Authors:** Dhanya Raj C. T, Vivekanandan Palaninathan, Surabhi Kandaswamy, Vimal Kumar, Rathinam Arthur James

**Affiliations:** a Department of Marine Science, Bharathidasan University, Tiruchirappalli, 620024, Tamilnadu, India; b Bio-nano Electronics Research Centre, Toyo University, 2100 Kujirai, Kawagoe, Saitama, Japan; c School of Pharmacy and Biomedical Sciences, University of Central Lancashire, Preston, Lancashire, PR1 2HE, UK

**Keywords:** Urolithiasis, Seaweeds, Oxidative stress, Inflammation, Nanoparticles, Nanomedicine

## Abstract

Urolithiasis affects a significant portion of the global population, causing discomfort and pain. Unfortunately, effective drugs to treat this disorder are currently unavailable due to multiple mechanisms and an incomplete understanding of its causes. Consequently, drugs with multiple targets could be a safer and more effective remedy for treating urolithiasis. Moreover, the current treatment options are expensive and come with a risk of complications and stone recurrence. Therefore, an alternative treatment that can prevent stone recurrence and reduce associated symptoms is necessary. Seaweeds are a rich source of beneficial metabolites, like antioxidants, anti-inflammatory, analgesic, and enzyme-inhibitory properties. Advances in nanotechnology hold great promise for improving the therapeutic potential of these metabolites. However, the use of nanoparticles for treating urolithiasis has yet to be explored well, and only a few reports exist on the use of terrestrial plant-based nanoparticles. This review examines the therapeutic properties of seaweed bioactive compounds and their possible applications in treating urolithiasis. We propose that seaweeds could be an excellent source of essential dietary minerals and other bioactive compounds with multiple targets to treat renal calculus naturally. Additionally, the review highlights the potential of nanomedicine in treating urolithiasis, proposing seaweed-based nanoparticles as a promising treatment option.

## 1 Introduction

The ocean is an incredible and significant part of our planet, covering a remarkable seventy-one percent of its surface and sheltering various marine organisms with diverse biological properties [1]. India's coastline spans about 7516.6 km and retains a total area of 3,287,263 km^2^, making it one of the countries with a rich marine ecosystem [2]. The nation is fortunate to possess diverse terrestrial and aquatic flora known for their therapeutic properties. Regrettably, these herbal plants are under threat of extinction due to overharvesting, loss of habitat, and climate change. Furthermore, cultivating and formulating active ingredients from herbal compounds in large quantities is challenging.

Fortunately, there is a solution to this challenge in the form of seaweed, which is easily accessible and abundant in India. They are an excellent source of essential dietary minerals and other bioactive compounds that show promise as drug candidates [3]. Several studies have explored their potential medicinal properties, and they have been used as folklore medicine for centuries in traditional medicinal systems worldwide. However, the wide medicinal application of seaweed is hindered by a lack of inherited knowledge about them and the unknown mechanism of action.

Several research studies have been conducted on seaweed to examine its active components and therapeutic benefits [3,4]. Seaweeds are easily accessible and are considered an invaluable asset. However, more research is needed to fully understand and utilize their vast potential in medicine and related fields. For instance, there are reports on studies that explore seaweed's anti-urolithiatic potential, which provides hope for the future. But, the mechanism of action behind this potential has yet to be explored.

This review explores the plausible mechanisms involved in urolithiasis and seaweed therapeutics, including the phytochemical constituents, biological activities, and their effect on ameliorating urolithiasis. The review also highlights the application of nanomedicine in urolithiasis, with proposed seaweed-based nanoparticles for urolithiasis treatment. The potential uses of seaweeds in treating various ailments, including urolithiasis, are vast and exciting. Seaweed's unique and promising drug candidates have the potential to revolutionize the field of medicine and offer a sustainable alternative to herbal plants.

Urolithiasis is a prevalent condition that needs an effective treatment. Nanomedicine presents a hopeful solution that can deliver drugs directly to the target site without compromising their properties. This method offers a significant advantage over conventional drug delivery systems. Additionally, nanoparticles have become increasingly popular due to their unique characteristics, such as small size, large surface area, high bioavailability, and minimal side effects [5,6]. Although no nanomedicine is currently available for urolithiasis, experts are investigating the use of bioactive compounds from seaweed combined with bio-fabricated nanoparticles as a possible treatment.

This is a promising field of research, and this review provides a comprehensive summary of the anti-urolithiatic potential of these compounds and their nanoparticles. This information is invaluable for researchers striving to develop seaweed-based anti-urolithiasis drugs.

## 2 Urolithiasis

Urolithiasis is a painful and concerning urological disorder affecting millions globally [7]. Its prevalence ranges from 1 to 15 %, with India reporting a particularly high prevalence rate of 12 %,and about 50 % of cases may lead to renal damage or kidney loss [8]. While urolithiasis can affect anyone, those between the ages of 21 and 60 are more susceptible [9]. Urolithiasis can lead to life-threatening complications such as chronic kidney disease and end-stage renal disease. The lifetime risk of developing urolithiasis varies considerably across regions, with the highest prevalence reported in Asia (1–19.1 %), followed by North America (7–13 %) and Europe (5–9%) [10]. Additionally, there is a 50 % risk of recurrence [11]. Diet and weather are the two primary factors determining the incidence, prevalence, recurrence rates, and constituent of calculi. High fat and sugar diets are associated with the high prevalence of urolithiasis in Asian countries [12]. Recent studies reported that the use of Vitamin C and D supplements as an anti-inflammatory treatment for COVID-19 potentially increases the prevalence of secondary hyperoxaluria, urolithiasis, and supplement-associated oxalate nephropathy cases, resulting in significant and irreversible acute kidney injury [[13], [14], [15]]. Men are three times more likely to develop urolithiasis than women, with testosterone acting as a promoter and estrogen acting as an inhibitor in stone formation [16].

Urolithiasis occurs when the urine is oversaturated with calculogenic substances, leading to the formation of calculi anywhere in the renal tract. Urinary stones are heterogeneous [17] and are categorized based on their chemical properties. These properties include acidic stones such as cystine and uric acid, neutral stones like calcium oxalate (CaOx), and alkaline stones such as magnesium ammonium phosphate (MAP). The most common type of renal stones is calcium-containing stones, with CaOx and calcium phosphate (CaP) making up 80 % of all cases. The remaining 20 % include struvite (10–15 %), uric acid (3–10 %), cystine (0.5–1%), and other types of stones [8]. Recurrent stone formation is a significant health problem associated with urolithiasis, which requires a better understanding of the disease's mechanism to prevent kidney stone recurrence and treatment.

### 2.1 Pathophysiology of urolithiasis

Kidney stones can develop due to various risk factors, ranging from oxalate metabolism to imbalances between lithogenesis-promoting and inhibiting factors, renal function, urine volume, urine pH, infection, diet, medication, genetic, and environmental factors (Table 1). Awareness of these risk factors is key to preventing the formation of stones. As such, it is imperative to understand and address these factors to maintain optimal health.

**Table 1:** Various factors contributing to kidney stone formation.

Risk Factors		Mechanism	Ref
**Urine composition**	Oxalate metabolism	Increased excretion of oxalate leads to hyperoxaluria which develops kidney stone	[18,19]
	Crystalloids-colloids imbalance	Increase in crystalloids (calcium, oxalate, cystine, uric acid) and decrease in colloids (mucin, sulfuric acid) levels leading to stone formation	[20]
	Decreased inhibitors and increased promoters	An imbalance between inhibitors (Magnesium, citrate, nephrocalcin, glycosaminoglycans, Tamm Horsfall protein, uropontin/osteopontin, pyrophosphate, bikunin, urinary trefoil factor 1, urinary prothrombin fragment 1) and promoters (uric acid, oxalate, calcium, phosphate, cysteine) may modify physiochemical conditions in urine leading to stone formation.	[21,22]
	Urine pH	High and low urinary pH may cause urine stone formation; Alkaline pH may promote CaP and struvite stone formation; Acidic pH may develop CaOx, cystein and uric acid stones	[23]
**Environmental Factors**	Hot and arid climate-dehydration, low water intake	Low urine volume leads to super saturation of salts and other minerals that leads to stone formation	[24]
**Genetic factor**	Hereditary	Family history; cystinuria, adenine phosphoribosyl transferase deficiency, Single gene disorders like distal renal tubular acidosis, Dent diseases, primary hyperoxaluria.	[25]
**Metabolic disorders**		Hyperoxaluria, hypercalciuria, hypocitraturia, hyperuricosuria, hyperparathyroidism, diabetic mellitus, hyperuricemia	[26]
**Congenital urinary anomalies**	Renal anatomy	Medullary sponge kidney, horseshoe kidney, ureteropelvic junction obstruction, autosomal dominant polycystic kidney disease	[27]
**Lifestyle Factors**	Infection	Hydrolysis of urea to ammonium by bacterial urease raises urine pH which forms MAP.	[28]
	Dietary factor	High intake of animal protein, sodium, calcium and oxalate and low intake of fluid results in hypercalciuria, uricosuria, oxaluria and low urine volume and pH which leads to urolithiasis.	[29,30]
	Medication	Some drugs, like indinavir sulfate, sulfonamides guaifenesin, triamterene, atazanavir and ceftriaxone, induce kidney stone formation by interfering with the metabolism of purine or CaOx. Furosemide decrease urinary volume and Sodium bicarbonate increase urinary calcium which favors stone formation	[31]

### 2.2 Mechanism of urolithiasis

Urolithiasis is a complex disease with metabolic syndrome associations that impact several biochemical pathways [8]. Although it has been known for some time, its molecular and physiological mechanism remains unclear. However, current understanding suggests that various physicochemical events occur in urothelial cells, including supersaturation of mineral components, crystal nucleation, growth, aggregation, and crystal retention [32].

**Fig. 1:**
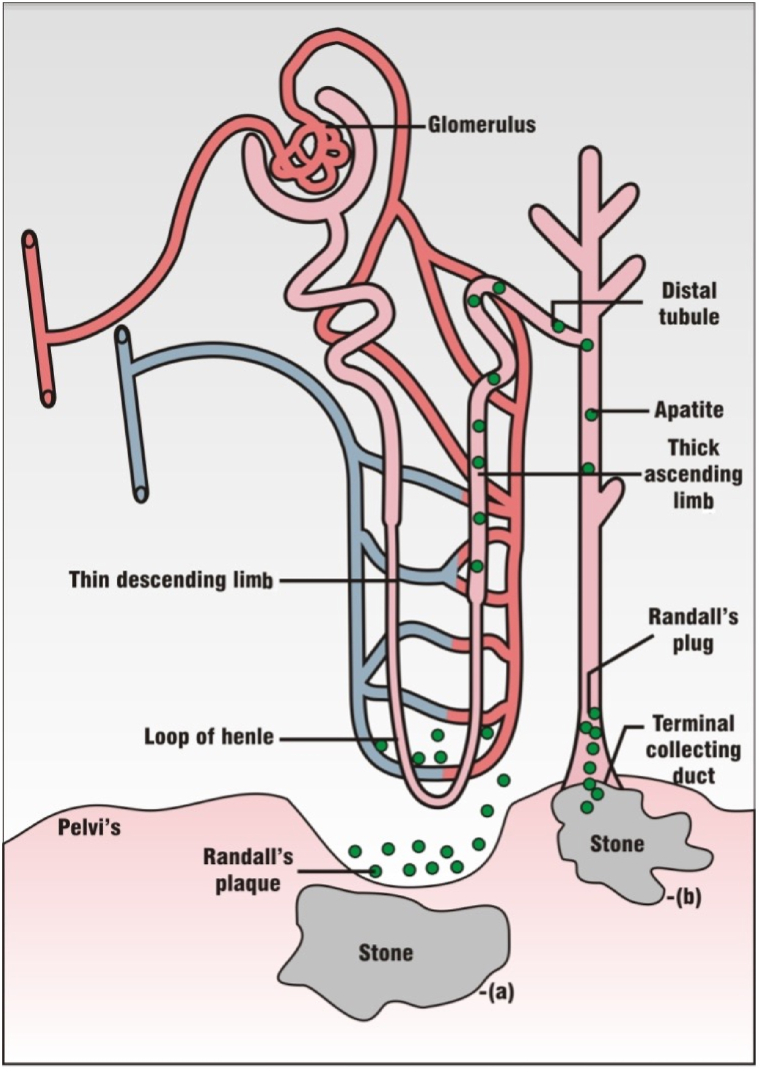
Two basic pathways (fixed-particle and free-particle mechanism) of stone formation. (a) Fixed-particle pathway: The crystals deposit in the interstitium, attach to the epithelial lining of the renal tubule, and form nidi for stone formation, (b) free-par particle pathway: The crystals form in the renal tubules travel through the duct of Bellini can grow, aggregate and deposit to the terminal collecting duct (Randall's plug) and become nidi for stone formation [34].

Exposure to any of the above-mentioned risk factors can alter concentrations of promoters and inhibitors. Increased concentrations of urinary stone promoters may cause hypercalciuria, hyperoxaluria, and hyperuricosuria, while reduced concentrations of urinary stone inhibitors may cause hypocitraturia, hypokaliuria, and hypomagnesiuria, leading to stone formation [33]. Thus, the disproportionate urinary stone promoters and inhibitors accompanied by low urine volume cause urine supersaturation, thereby triggering the nucleation process. Nucleation occurs when crystalloids merge to form tiny particles (>10 nm) in the renal tubule lumens or the Henle loop basement membranes through the free particle pathway or the fixed particle pathway or both as depicted in (Fig. 1) [34]. These crystal nuclei grow and aggregate, reaching pathological sizes (tens of microns) that can be toxic and injure renal epithelial cells. Injured tubules provide a suitable site for crystal adherence and retention, resulting in nidus (stone nucleus) formation that grows to form a stone ranging in size from millimeters to several centimeters [35]. Therefore, comprehending the risk factors and their impact on the balance of promoters and inhibitors is vital in preventing stone formation.

**Fig. 2:**
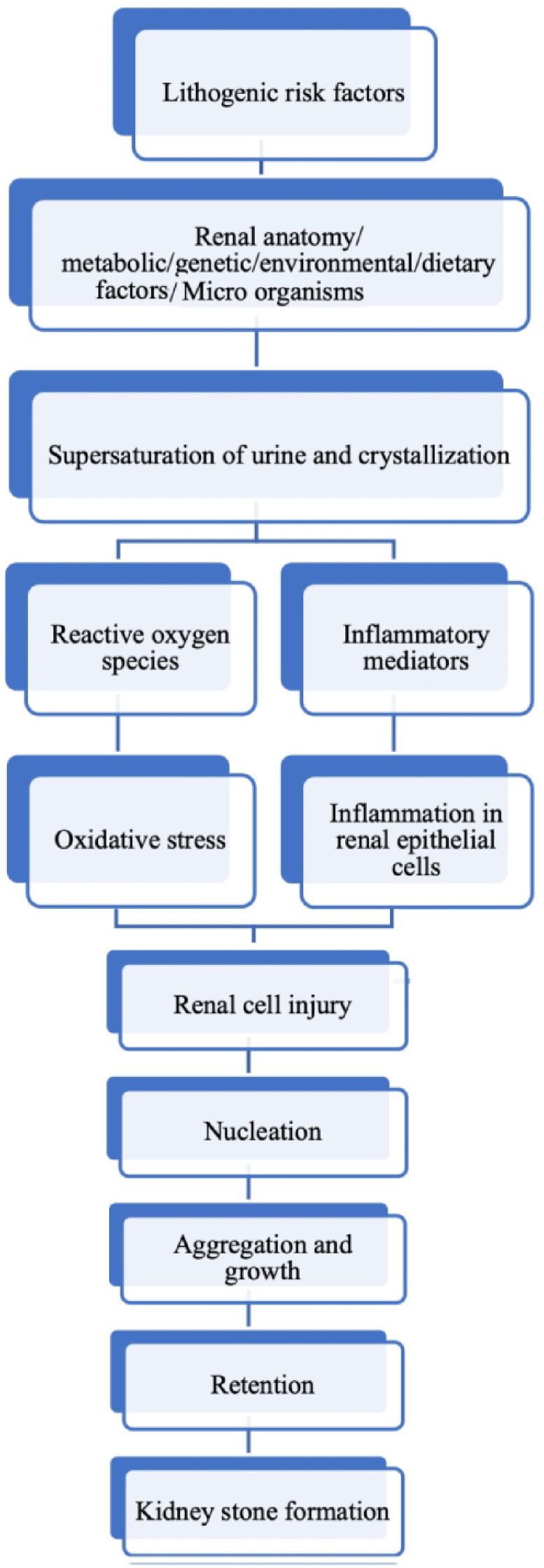
Mechanism of pathophysiology of urolithiasis.

The development of stones in the kidneys can be attributed to various factors, including inflammation and oxidative stress. When an excess of crystals is present, reactive oxygen species (ROS) are produced in renal cells, leading to further damage. Additionally, the deposition of crystals encourages the release and expression of inflammatory mediators (cytokines, chemokines, growth factors, and enzymes), promoting inflammatory responseleading to renal tubular injury and cell death. As a result, proteins and dead or damaged cells are shed and released into the urine (Fig. 2). provides a detailed illustration of how these factors amplify crystal formation, their growth or aggregation, retention, and ultimately the formation of kidney stones [33].

#### 2.2.1 Oxidative stress in urolithiasis

An imbalance between oxidants and antioxidants in the body can lead to an oxidative stress phenomenon, which causes the overproduction of reactive oxygen species (ROS). These ROS are oxygen-containing free radicals, atoms, or molecules with one or more unpaired electrons that can harm cells and tissues. Some common examples of cellular ROS include superoxide anion (O_2_^•−^), hydroxyl radical (^•^OH), peroxides, nitric oxide radical(^•^NO), alpha oxygen, and singlet oxygen. Most of these ROS are produced as by-products of various cellular processes, including mitochondrial electron transport, endoplasmic reticulum oxidoreductase enzymes, and NADPH oxidase (nicotinamide adenine dinucleotide phosphate oxidase) [36]. They are highly reactive signaling molecules that play an essential role in the cell cycle, immune regulation, redox balance, inflammation, and stress-related apoptosis. It alters the oxidant-antioxidant balance in kidney cells, resulting in injury and inflammation during urolithiasis [37]. They can also react with cell constituents like proteins, carbohydrates, lipids, and nucleotides, making modifications like promoting lipid peroxidation, DNA damage, and protein oxidation and carbonation [38,39]. It is worth noting that in mitochondria, reactive oxygen species (ROS) are produced as a by-product during a typical electron transport chain reaction. This occurs more significantly in the kidney compared to other tissues. Under normal conditions, the production of ROS in the body is regulated by various antioxidants and scavengers in the cell. The kidneys contain two major sources of ROS, namely mitochondria and NADPH oxidase [40]. When exposed to high quantities of crystals such as oxalate, CaOx, CaP, and uric acid, the rennin angiotensin system (RAS) gets activated, leading to an increase in rennin levels and the generation of angiotensin II. This, in turn, activates NADPH oxidase, which triggers the production of ROS in the kidneys [41]. The crystals can also affect various proteins that play a role in energy production, which can cause mitochondrial dysfunction and further increase ROS production [42]. Excessive production of ROS and reduction in antioxidant levels can lead to an imbalance in the oxidant-antioxidant system. This imbalance can cause oxidative stress, which can disrupt the control of redox signaling and ultimately damage biomolecules, leading to cell injury [33]. For instance, the oxidants can react with lipids, carbohydrates, nucleic acids, and proteins in the cells and perturb their structural and functional characteristics, which causes cell membrane injury that facilitates the retention of crystals, which forms kidney stones.

#### 2.2.2 Inflammation in urolithiasis

Direct contact with crystals can harm renal tubular epithelial cells. Such contact triggers an inflammatory response by prompting the production and release of inflammatory mediators, leading to tubular injury. The NOD-LRR-and-pyrin domain-containing protein3 (NLRP3) is involved in the crystal-induced inflammatory response by activating and assembling NLRP3 inflammasomes. The NLRP3 inflammasome is a large cytoplasmic protein complex that regulates the inflammatory response through interleukin-1β (IL-1β).

The crystal-induced inflammation pathway mechanism involves several steps. First, it generates ROS. Second, crystals are taken up into intracellular lysosomes, which lead to overloading and lysosomal leakage, which activates NLRP3-mediated inflammasome assembly [43]. NLRP3 activation triggers the secretion of the potent proinflammatory cytokine IL-1 β. Crystals also stimulate the release of danger signals, activating Toll-like receptors (TLRs) and IL-1 receptors. These activated receptors trigger NLRP3 inflammasome activation, releasing IL-1 β, which then matures and sets up subsequent inflammation and injury [[43], [44], [45]]. Therefore, oxidative stress and inflammation induce the shedding of cell debris and the release of various proteins into the urine, creating a favorable environment for stone formation.

#### 2.2.3 Infection in urolithiasis

Certain urease-producing organisms, such as *Proteus*, *Pseudomonas*, or *Klebsiella*, can cause infections in the upper urinary tract (UTI). These infections can result in the development of MAP stones and carbonated apatite. When a UTI occurs, the microorganisms produce urease, breaking urea into ammonia and carbon dioxide (CO_2_). These substances then react with water to form ammonium and carbonic acid, as shown in equations (1), (2)).(1)(2)

The urine pH can increase from 7.2 to 8 when hydroxide reacts with weakly acidic carbonic acid. This alteration can harm the glycosaminoglycan layer in the renal tubule, making it susceptible to bacterial adhesion and biofilm formation [46]. Furthermore, in alkaline conditions, magnesium precipitates and becomes insoluble, while ammonium combines with naturally occurring urinary cations, such as magnesium, calcium, and phosphate, to create a hard coating on the biofilm surface, eventually forming a stone. It's important to note that MAP forms at a pH of 7.2 or higher, while carbonate apatite develops betweenpH 6.8 and 7.2 [47].

### 2.3 Current treatment

Small kidney stones that measure up to 5 mm in diameter typically do not require medical intervention. In such cases, doctors may suggest pain medication to help alleviate any discomfort until the stone passes on its own [48]. However, larger stones, particularly those that exceed 7 mm, can lead to serious health issues, including severe pain, burning during urination, urinary tract obstruction, and infection [49], and may require surgical intervention [50]. These surgical treatments, such as shock wave lithotripsy and endoscopic management, may result in adverse side effects and do not eliminate the risk of kidney stone recurrence [51]. Unfortunately, there is no effective allopathic medication for urolithiasis. The available drugs, such as diuretics and narcotic analgesics, have limited efficacy and unclear mechanisms of action [52]. Long-term use of these drugs may increase the risk of adverse drug effects and long-term fertility problems, with no guaranteed cure [53].

The development of effective drugs for urolithiasis has proven to be challenging due to the diverse pathophysiological mechanisms and causes of the condition [54]. So, to address this issue, drugs with multiple targets have become a more attractive option. For instance, plant extracts containing phytochemicals can prevent stone formation by acting through several pathways, including diuretic, crystallization inhibition, lithotriptic, analgesic, anti-inflammatory, antioxidant, and regulation of oxalate metabolism [[55], [56], [57], [58]]. Likewise, seaweeds, on the other hand, are rich in bioactive substances that have been shown to impact human health in multiple ways, as shown by research in marine chemical ecology [59]. Further studies have been carried out on seaweeds to explore their medicinal properties, which will be discussed in subsequent sections.

## 3 Seaweeds- A promising therapeutic agent in urolithiasis

The scientific community has always been fascinated by the abundance of the ocean, particularly with regard to natural product exploration in marine organisms. Seaweeds are abundant in nature, but unfortunately, they have not been utilized to their fullest potential. For centuries, seaweed has provided various uses, from being a dietary staple and animal feed to a source of pharmaceutical drugs, especially in Asia [60]. Seaweeds are macroalgae that thrive in marine and brackish water environments and are categorized into Chlorophyta (green algae), Phaeophyceae (brown algae), and Rhodophyta (red algae) based on their pigments. Chlorophyta contains chlorophyll *a* and *b*, Phaeophyceae contains fucoxanthin, and Rhodophyta contains phycoerythrin [61]. Seaweeds are typically found in shallow rocky coastal areas, particularly in low tidal regions. With over 10,000 species of marine algae worldwide, India alone has reported about 740 species [62]. Seaweeds are rich in bioactive compounds that hold great promise and have significant applications in promoting human and animal health, making them a promising area for future research.

### 3.1 Bioactive compounds in seaweeds

Seaweeds are highly valued for the wide array of bioactive compounds they contain, which offer numerous therapeutic benefits. These compounds include proteins, polysaccharides, lipids, vitamins, carotenoids, enzymes, minerals, essential fatty acids, antibiotics, dietary fiber, and many other valuable chemicals [63]. Scientific research has revealed that these compounds possess a range of beneficial properties, including antibacterial, antifungal, antiviral, antineoplastic, antifouling, anti-inflammatory, antitumor, cytotoxic, and antimitotic activities, making seaweeds a highly sought-after subject of study [64,65].

The remarkable therapeutic potential of seaweeds has led to a growing interest in their medicinal properties. Table 2 provides a comprehensive list of experimental studies that have explored the medicinal benefits of seaweeds. Given their many advantages, it is no surprise that seaweed research continues to gain popularity as a promising source of natural remedies.

**Table 2:** Experimental studies on seaweeds and their medicinal properties.

Seaweed	Classification	Properties	Bioactive compound	Ref
***Ulva lactuca***	Chlorophyta	Anti-inflammatory	3-O-β-D glucopyranosyl-stigmasta-5,25-dien	[66]
***Codium iyengarii***	Chlorophyta	Moderate antibacterial	Steroidal glycosides	[67]
***Eucheuma striatum***	Rhodophyta	COM crystals inhibition	Sulfated Polysaccharides	[68]
***Fucus spiralis***	Phaeophyceae	Antioxidant activity	polymeric phenols	[69]
***Fucus vesiculosus***	Phaeophyceae	Diuretic activity	Fucoidan	[70]
***Sargassum fulvellum, Sargassum thunbergii***	Phaeophyceae	Antipyretic, analgesic and anti-inflammatory activity	Dichloromethane, ethanol, and boiling water extract^a^	[71]
***Colpomeniasinuosa***	Phaeophyceae	Antioxidant activity	Dimethyl sulfoxide and methanol extracts^a^	[72]
***Sargassum* sp.**	Phaeophyceae	Antioxidant and antimicrobial activity	Methanol extract^a^	[73]
***Padina boergesenii***	Phaeophyceae	Diuretic activity, anti-urolithiatic activity	Ethanolic extract^a^	[74]
***Rhodomelaconfervoides***	Rhodophyta	Antioxidant activity	Methanol–chloroform extract^a^	[75]
***Acanthophoraspicifera*, *Padinatetrastromatica*, *U. lactuca***	Rhodophyta, Phaeophyceae, Chlorophyta	Antioxidant activity	Methanol and water extract^a^	[76]
***F. vesiculosus*, *Fucus serratus*, *Ascophyllum nodosum*, *Pelvetiacanaliculata***	Phaeophyceae	Antioxidant activity	Phenolic compounds	[77]
***A. nodosum***	Phaeophyceae	Antioxidant activity	Phenolic compounds	[78,79]
***Callophycus serratus***	Rhodophyta	Antifungal	Bromophycolides	[80]
***Dichotomariaobtusata***	Rhodophyta	Analgesic and anti-inflammatory activity	Aqueous extract^a^	[81]
***Sargassum swartzii, Ulva reticulate***	Phaeophyceae, Chlorophyta	Analgesic and anti-inflammatory activity	Methanolic extracts^a^	[82]
***Laurencia glandulfera*, *Hypneamusciformis*, *Cystoseirabarbata*, *Sargassum vulgare*, *Enteromorpha compressa*, *Chaetomorpha linum*, *Cystoseiraericoidies*, *Sacchorizabulbosa*, *Corralina officinalis*, *Geliduimsesquipedale***	Rhodophyta, Rhodophyta, Phaeophyceae, Phaeophyceae, Chlorophyta, Chlorophyta, Phaeophyceae, Phaeophyceae, Rhodophyta, Rhodophyta	Anti-inflammatory and analgesic	Methanolic extract^a^	[83]
***Gracilaria* Sp.**	Rhodophyta	Spermicidal, anti-implantation, antimicrobial, antihypertensive, antioxidant, anti-inflammatory, Analgesic	Extract^a^	[84]
***Turbinariaconoides*, *Padina gymnospora*, *Sargassum tenerrimum***	Phaeophyceae	Antimicrobial	Methanol extract^a^	[85]
***Codium fragile***	Chlorophyta	Antipyretic, analgesic and anti-inflammatory activity	dichloromethane extract^a^	[86]
***Sargassum graminifolium***	Phaeophyceae	Anti-urolithiatic, inhibit CaOx crystallization	Sulfated Polysaccharides	[87]
***F. vesiculosus***	Phaeophyceae	Antioxidant activity	Phlorotannins	[88]
***P. gymnospora*, *Hypneamusciformis*, *Ulva fasciata*, *Caulerpa prolifera***	Phaeophyceae, Rhodophyta, Chlorophyta, Chlorophyta	Antibacterial	Ethanol, methanol, hexane, acetone-based extracts^a^	[89]
***Sargassum muticum***	Phaeophyceae	Antioxidant, antiproliferative, and anti-angiogenesis activity	Polyphenol	[90]
***Sargassum ilicifolium***	Phaeophyceae	*Anti-inflammatory and analgesic*	*Methanolic extracts*^a^	[91]
***Dictyopterisjustii***	Phaeophyceae	*Antioxidant, CaOx crystallization inhibition*	*Sulfated Polysaccharides*	[92]
***Caulerpa racemosa***	Chlorophyta	Antinociceptive, anti-inflammatory Activities	Sulfated Polysaccharides	[93]
***Gracilaria dura***	Rhodophyta	Diuretic activity	Methanol extract^a^	[94]
***Cystoseira*sp**	Phaeophyceae	Antioxidant, anti-inflammatory, antiproliferative	Aqueous extract^a^	[95]
***Cystoseiranodicaulis*, *Himanthaliaelongata*, *F. serratus*, *F. vesiculosus***	Phaeophyceae	Antioxidant activity	Phenolic compounds	[96]
***Porphyra vietnamensis***	Rhodophyta	Analgesics, anti-inflammatory, antioxidant and antiulcer properties	Aqueous and alcoholic fractions	[97,98]
***Caulerpa scalpelliformis***	Chlorophyta	Antiurolithiatic	Ethyl alcohol extract	[99]
***Saccharina japonica*, *Sargassum horneri***	Phaeophyceae	Antioxidant activity	Fucoxanthin	[100]
***S. vulgare*, *Sargassum fusiforme***	Phaeophyceae	Antimicrobial	Diethyl ether and ethanol extracts^a^	[101]
***Ulva armoricana***	Chlorophyta	Anti-viral, anti-oxidant	Sulfated galactans	[102]
***Ulva pertusa***	Chlorophyta	Anti-inflammatory	3-Hydroxy-4,7-megastigmadien-9-one	[103]
***Laminaria digitata*, *Undariapinnatifida***	Phaeophyceae	Antifungal activity	Crude extracts	[104]
***Eucheuma cottonii***	Rhodophyta	Analgesic	Sulfated Polysaccharides, terpenes, and peptides	[105]
***Laminaria japonica***	Phaeophyceae	Antioxidant activity, anti apoptoptic	Sulfated Polysaccharides; Fucoxanthin	[106,107]
***Ecklonia cava***	Phaeophyceae	Anti-neuroinflammatory, antioxidant, anti-inflammatory, immunomodulatory activities	Phlorotannins	[[108], [109], [110]]
***Enteromorpha prolifera***	Chlorophyta	*Anti-diabetic activity*	*Flavonoids*	[111]

a Bioactive compound undetermined.

### 3.2 Applications of seaweeds in urolithiasis treatment

#### 3.2.1 Seaweeds as diuretics

Insufficient water intake and degradation are potential causes of low urine output. In a healthy individual, the average 24-h urine output is around 1300 mL per day [112]. However, low urine volume can significantly increase the concentration of minerals such as calcium, oxalate, phosphorus, uric acid, salt, and other minerals, leading to the formation of kidney stones [113].

It is important to note that due to global warming, the incidence of kidney stone formation is expected to increase by 40–56 % by 2050 [114]. The pH level of urine is a significant factor in the development of kidney stones, as it promotes the formation of solid crystal phases such as CaOx or CaPand also affects the solubility of these stones. The normal pH range of urine over 24 h varies from 4.4 to 8, depending on different physiological factors [23], with an average of 6.0 (slightly acidic) [115]. Different kidney stones can form depending on the pH levels [116]. For instance, alkaline or high urine pH can promote the formation of CaP and magnesium ammonium phosphate (MAP) calculi, while acidic pH can increase the risk of CaOx, uric acid, and cystine stones [23]. Consumption of acid-ash foods decreases the urinary pH, whereas alkaline-ash foods increase the urinary pH. Increased consumption of animal proteins with high purine content and sulfur-containing amino acids can lower urine pH and increase the risk of uric acid stone formation. Conversely, high citrate content can increase urine pH and citrate excretion, which increases the risk of CaP stones [113].

Increasing urine volume and dilution is one effective way to manage urinary calculi. Diluting urine can effectively decrease the bacterial load and crystal precursor concentration, ultimately reducing stone size and promoting excretion. Moreover, this process can indirectly help clean inflammatory deposits [117]. Diuretics are medications that increase urine production and sodium excretion, and they can also regulate the volume and composition of body fluids. Seaweeds are a great source of bioactive compounds that have diuretic effects, which can help decrease body water [118]. However, most diuretic medications have side effects such as impotence, fatigue, and weakness. Recent studies on *P. boergesenii* (Phaeophyceae) [74] and *G. dura* (Rhodophyta) [94] have confirmed their diuretic properties.

#### 3.2.2 Seaweeds as an antioxidant agent

Antioxidants protect cells from damage caused by free radicals, or ROS, by either scavenging or deactivating them. Free radicals, or ROS, are formed when renal cells are exposed to lithogenic crystals and oxalate. Free radicals are highly reactive and unstable because they contain an unpaired electron. Natural antioxidants prevent cellular damage by donating an electron or hydrogen atom to the free radical. High levels of ROS and low levels of antioxidants in a cell can cause oxidative stress, which alters cell membrane integrity and oxidizes proteins and lipids, ultimately leading to cell rupture. Recent studies have shown that antioxidant treatment can reduce kidney injuries caused by crystals by maintaining the normal concentration of ROS, protecting endothelial cells from oxidative damage [119]. Antioxidants such as Vitamin E and ascorbic acid have shown potential in treating urolithiasis [120]. Therefore, treatment with antioxidants and free radical scavengers can significantly reduce the recurrence of renal calculi and cell damage.

Seaweeds contain many bioactive compounds with antioxidant properties, such as phenolic compounds, fatty acids, carotenoids, sulfated polysaccharides (SPs), peptides, vitamins, and sterols. Fucoidan and heparin, two low molecular weight polysaccharides, have been shown to protect against oxalate-induced oxidative renal injury. Specifically, fucoidan has been observed to inhibit the generation and subsequent release of ROS, while also effectively clearing these harmful molecules [121]. Further,SPs from *S. graminifolium* (Phaeophyceae) have also been found to have antioxidant properties and can inhibit CaOx crystallization [87]. SPs can also reduce ROS production and oxidative stress, enhance cell membrane repair mechanisms by increasing ATPase activity, protect and restore renal cells by impeding inflammation and injury pathways, increase free-radical scavenging activity, and prevent crystal aggregation and retention in the renal epithelial tissue. SPs are also known to have the remarkable ability to minimize the production of ROS and oxidative stress. This, in turn, assists in managing lipid-protein oxidation. Furthermore, SPs effectively enhance the cell membrane repair by elevating the ATPase activity. Additionally, SPs offer protection and restoration to renal cells by impeding inflammation and injury pathways while strengthening the free-radical scavenging activity of cells. Ultimately, this helps to prevent the buildup of crystals and their retention in renal epithelial tissue [122].

#### 3.2.3 Seaweeds as an anti-inflammatory agent

When the body encounters infections, injuries, or harmful agents, it activates a natural defense mechanism known as inflammation. Biochemical mediators like histamine, serotonin, peptides such as bradykinin, eicosanoids like prostaglandins, leukotrienes, and thromboxanes, platelet-activating factor, proinflammatory cytokines, fibrin, complement component C3, nitric oxide, and others are responsible for regulating the inflammation process. These mediators increase vascular permeability by vasodilation, allowing immune cells to migrate to the injury site. Inflammation often manifests in symptoms such as pain, redness, warmth, and swelling.

Urolithiasis is a condition caused by excessive ROS secretion, leading to oxidative stress and inflammation in kidney epithelial cells. It may be possible to alleviate the symptoms of urolithiasis by inhibiting inflammation. Anti-inflammatory allopathic drugs could be used to inhibit the inflammation around the injured site, but they may have potential side effects. In this scenario, seaweeds can be used as natural alternative to allopathic drugs as they are a great source of immune-modulatory compounds such as fatty acids, terpenes, bioactive peptides, and polysaccharides. More so, SPs are the main componentsof seaweeds that possess anti-inflammatory properties. Some examples of SPs found in seaweeds are sacran [123,124], fucan [125], and fucoidan [126]. These compounds can interfere with many pathways in the immune and inflammatory systems, making them effective anti-inflammatory agents. Other bioactive compounds found in seaweeds that have been studied for their anti-inflammatory activity include polyphenols, phlorotannins [127], fucoxanthin [128], phenolic compounds such as catechol, rutin, and hesperidin [129], diketopiperazine [130], and alkaloids [131].

#### 3.2.4 Seaweeds as phospholipase A2 enzyme inhibitor

Phospholipase A2 (PLA_2_) is a highly active enzyme that plays a major role in causing cellular injury. Its mechanism of action involves hydrolyzing phospholipids andcausing free fatty acid accumulation, such as arachidonic acid, lysophospholipids, and ceramide. These free fatty acids can trigger ROS production in mitochondria and cause renal cell injury or cell death [132]. Enzymes like cyclooxygenase or 5-lipoxygenase then metabolize the free fatty acids, producing eicosanoids (prostaglandins, thromboxanes, and leukotrienes) and platelet-activating factors, which are the primary inflammatory mediators in urolithiasis that promote acute inflammation. According to a study [133], monoglycerides from *Sargassum* *sagamianum* (Phaeophyceae) effectively inhibited PLA_2_ and cyclooxygenase-2 activity. Other bioactive compounds, such as alginates, fucoxanthin, and phlorotannins, have also shown effective lipase inhibitory activity.

#### 3.2.5 Seaweed as an analgesic agent

Pain is a discomforting sensation resulting from physical injury, illness, or emotional stress. To alleviate pain and reduce inflammation, analgesics are commonly prescribed. These medications target pain-causing mechanisms in the central or peripheral nervous system while preserving consciousness [134]. Analgesics can be classified as corticosteroids or non-steroidal compounds such as aspirin. Nonetheless, the use of either type of drug can potentially lead to toxic effects or other adverse reactions.

The common signs and symptoms of disorders caused by kidney stones include pain in the back and side that spreads to the lower abdomen, blood in urine, frequent urination, pain during urination, nausea, and vomiting. Kidney stones come in various sizes and have rough or sharp edges. Small stones can pass through the urine without any issues. However, large stones can be extremely painful while passing through the urinary tract.

Individuals who suffer from urolithiasis may encounter an abrupt and excruciating pain that initiates in the abdomen and appears and disappears in waves. This discomfort can radiate to other regions, such as the groin, testis, or vulva, and can be extremely severe and may last for 20–60 min. This particular type of colicky-typepainis referred to as renal colic, which is triggered by a range of pain receptors, including prostaglandins. Prostaglandins stimulate the generation of cyclic adenosine monophosphate in sensory neurons, which heightens the pain [135]. Further, they also activate voltage-gated sodium channel type v1.8 receptors, which can amplify the discomfort even more.

There are several plant compounds that are known for their analgesic effects, including flavonoids, volatile oils, phenol compounds, alkaloid compounds, organic acids, and essences. These compounds work by reducing the activity of cyclooxygenase-2 and preventing the formation of prostaglandins at the site of pain. Flavonoids, in particular, have been found to cross the blood-brain barrier and modulate pain centrally through different mechanisms, such as affecting opioid GABA, alpha 2 adrenergic and inhibiting the enzymes involved in inflammation. For instance, tarragon is one such plant that contains flavonoids and other compounds that decrease pain by protecting against oxidative stress caused by hyperglycemia and having properties similar to benzodiazepines [136].

Research has shown that seaweeds contain compounds with analgesic properties, making them a promising source of pain relief. In fact, a study by Vázquez et al. [137] found that *D. obtusata* (Rhodophyta) contains lactones, phenols, triterpenes, steroids, and reduced carbohydrates that have the potential to act as analgesics. Additional studies on seaweeds like *S. swartzii* (Phaeophyceae) and *U. reticulate* (Chlorophyta) [82] *C. fragile* (Chlorophyta) [86], and *P. vietnamenis* (Rhodophyta) (98) have demonstrated their efficacy in relieving pain-related symptoms.

#### 3.2.6 Seaweeds as an anti-apoptotic agent

The interaction between crystals and cells is a crucial step in kidney stone formation. Exposure of renal epithelial cells to oxalate or hyperoxaluria results in oxalate DNA damage in both nuclear and mitochondrial DNA and altered gene expression, which ultimately triggers the activation of the p38 mitogen-activated protein kinase (p38 MAPK) signaling pathway and leads to renal tubular cell apoptosis [8].

Recent research has revealed the anti-apoptotic properties of fucoxanthin, a bioactive ingredient present in brown seaweed *L. japonica*, in different cell types. This effect is achieved through scavenging ROS, inducing heme oxygenase-1, and activating the phosphoinositide 3-kinase (PI3K/Akt) signaling pathway. Fucoxanthin increases the expression of the transcription factor PPARα, which leads to NHE1 upregulation, further activating the PI3K/Akt signaling pathway and preventing apoptosis [107].

Fucoidan, present in various seaweeds such as *F. vesiculosus* (Phaeophyceae) [68] and *L. japonica* (Phaeophyceae) [138], has been found to have a renoprotective effect. It reduces lipid peroxidation and mitochondrial swelling, normalizes cell membrane integrity and leakage of enzymes, improves the antioxidant status of damaged renal tissue, and prevents renal apoptosis [139].

#### 3.2.7 Seaweeds as an antimicrobial agent

Patients who suffer from kidney stones are more susceptible to developing kidney infections, and at times, urinary tract infections themselves can result in the formation of stones. Some species of seaweed contain bacteriostatic or bacteriocidal compounds that can inhibit the growth of some pathogenic bacteria. These compounds have the potential to be utilized in treatments for bacterial infections. For instance, seaweeds possess amino acids, terpenoids, phlorotannins, acrylic acid, phenolic compounds, steroids, halogenated ketones and alkanes, cyclic polysulphides, and fatty acids, which are capable of inhibiting the growth of pathogenic bacteria [140]. Several studies have demonstrated that some seaweeds exhibit antibacterial properties that can effectively combat UTI bacteria like *Escherichia coli, Klebsiella pneumoniae,* and *Proteus mirabilis*. For example, Di-isooctylphthalate, an aromatic ester derivative from *U. lactuca*, has been found to possess significant antibacterial activity against UTI bacteria. This compound reduces the activity of virulence genes in the bacteria, thereby preventing or mitigating their resistance and pathogenicity [141]. Numerous research studies have demonstrated the antimicrobial activity of diverse seaweeds against UTI bacteria [[142], [143], [144]]. These findings support the potential use of seaweeds as a natural alternative to conventional antibiotics for treating UTIs.

### 3.3 Antiurolithiatic activity of seaweeds

Research on seaweeds has revealed that their SPs and phenolic compounds possess biological activity [145]. SPs are negatively charged due to sulfate, hydroxyl, and carboxyl groups and play a crucial role in inhibiting the formation of calcium oxalate (CaOx) kidney stones [146]. The physiological mechanism by which SPs exert their inhibitory effect on CaOx crystallization involves several steps. Firstly, negatively charged SPs directly interact with growing calcium crystals, modifying their surface charge density and blocking their growth sites by either coating the crystal surface or changing its properties. Secondly, SPs decrease the concentration of CaOx in supersaturated urine by binding to calcium ions, thereby reducing nucleation. Thirdly, SPs prevent renal stone formation by suppressing the amount of promoters of the stone formation while enhancing urinary inhibitory activity. Finally, SPs stabilize calcium oxalate dehydrates (COD) crystals that are thermodynamically unstable, thereby preventing them from transforming into more stable calcium oxalate monohydrate (COM) crystals by increasing the negative value of the zeta-potential, which reduces the possibility of CaOx stone formation [92]. Phenolic compounds also act as anti-crystalline agents by creating soluble chemical species that can reduce/prevent the crystallization process. They also bind directly to crystalline surfaces due to their negative charge and inhibit further growth and aggregation of crystals. Moreover, phenolic compounds eliminate free radicals, prevent oxidative stress, and act as diuretics, flushing out minute crystals through urine [147]. Several studies have demonstrated the effectiveness of different seaweed extracts and isolated SPs on CaOx crystallization using diverse crystallization models with various media.

In a study by Vasanthi et al., the antiurolithiatic activity of alcoholic extracts of *P. boergesenii* was evaluated in adult Wistar albino rats with hyperoxaluria [74]. The results indicated that the ethanolic extract of *P. boergesenii* (Phaeophyceae) significantly reduced calcium and oxalate excretion at a dose of 150 and 200 mg/kg. The extract also demonstrated the ability to decrease the supersaturation of urinary CaOx oxalate, increase urine volume, decrease urinary risk factors, and prevent further damage to the renal tubules. These findings suggest that *P. boergesenii* has potential diuretic and antiurolithiatic properties. However, further exploration of the active metabolites present in *P. boergesenii* is necessary to understand its mechanism of action better.

Lekha et al. studied the inhibitory effect of marine halophytes, including seagrasses (*Syringodiumisoetifolium* and *Cymodoceaserrulata*) and seaweeds (*Dictyotadichotoma* (Phaeophyceae), *Stoechospermum marginatum* (Phaeophyceae)*, Sargassum wightii* (Phaeophyceae)*, C. scalpelliformis* (Chlorophyta), and *Valaniopsis pachynema* (Chlorophyta)), on CaOx crystal formation [99]. The study revealed that the ethyl alcohol extract of *C. scalpelliformis* showed the maximum CaOx crystal inhibition (83.34 ± 0.015 %), comparable to the inhibition effect (91.68 ± 0.02 %) of the standard drug Cystone. The results suggest that *C. scalpelliformis* contains potential molecular compounds that can inhibit the formation of CaOx crystals. Further research could identify the lead compound responsible for the inhibitory effect. In another study, Santana et al. investigated the antioxidant, anti-urolithiatic, and anti-proliferative properties of the crude extract of *Gracilaria birdiae* (Gb) (Rhodophyta) collected from three different sources (GbD from drift, GbNB from the natural bank, and GbF from algae cultivation). While GbF exhibited higher antioxidant activity than GbD and GbNB, the crude extract of GbF increased the formation of smaller size and less aggressive COD crystals. Furthermore, all three extracts showed noncytotoxic and nonproliferative effects on human kidney cells and HELA cells, respectively [148].

In a study conducted by Ouyang et al., the inhibitory effects of degraded and natural SPs from *L. japonica* (LPS) (Phaeophyceae) on CaOx crystallization were explored [52]. The results showed that degraded and natural LPS can inhibit CaOx crystallization, but degraded LPS exhibited a higher inhibition rate than natural LPS. This is because degraded LPS can better inhibit CaOx crystal growth and induce COD formation than natural LPS. Further investigation is needed to understand the molecular mechanisms underlying crystallization inhibition, both *in vivo* and *in vitro*, to ensure the safe use of LPS for human-related applications. Zhang et al. [87] conducted a study that revealed the potential of polysaccharides extracted from *S. graminifolium* (Phaeophyceae) to prevent the crystallization of CaOx. Extensive research has been conducted on the inhibitory effect of *S. graminifolium*SPs on CaOx crystallization and its impact on crystal morphology. Additionally, their findings from electrical conductivity studies suggest that higher concentrations of SPs result in a more prominent inhibition of CaOx crystal. They attributed the ability of SPs to inhibit crystallization to the presence of carboxyl, hydroxyl, and sulfuric acid in the compound [149]. This study suggests that SPs from *S. graminifolium* could be a potential therapeutic agent for further clinical investigations. Similarly, Gomes et al. evaluated the effect of SPs from *Caulerpa cupressoides* (Chlorophyta) (CCB-F0.3, CCB-F0.5, CCB-F1.0, and CCB-F2.0) on CaOx crystallization *in vitro* and described the various morphological characteristics of the formed CaOx crystals. They proposed a model of interaction between the populations of SPs obtained within the CaOx crystals [150]. In another study conducted by Oliveira et al. [151], a sulfated galactan was extracted from a red seaweed, *G. birdiae*. After purification, the galactan's potential as an antioxidant and anti-urolithiatic agent was evaluated. The results confirmed that *G. birdiae* galactan (GB) possessed antioxidant properties, which were demonstrated through various assays. When present, GB increased the number of CaOx crystals while decreasing their size, indicating that it stimulated nucleation and inhibited aggregation. This nucleation stimulation led to more nuclei formation through the consumption of CaOx, which eventually forms crystals until the concentration reaches below the saturation level. GB also disrupted the crystalline network, forming more amorphous rounded COM and COD crystals. Additionally, GB stabilized the COD found in the urine of healthy individuals and reduced CaOx stone formation by modifying the zeta potential of crystals. The researchers noted that GB had a non-cytotoxic effect on human kidney cells (HEK-293). These findings suggest that GB could be a safe and potential candidate for treating urolithiasis.

## 4 Seaweed-based nanomedicine in urolithiasis management

**Fig. 3:**
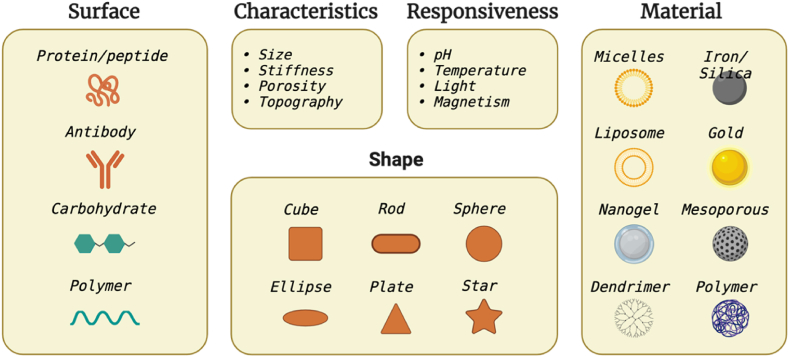
Different traits and factors nanoparticles offer for biomedical applications to deliver drug/gene/protein (Created with BioRender.com).

Nanomedicine is a growing area of research at the intersection of nanotechnology and medicine, with promising potential in the healthcare industry. This interdisciplinary field involves manipulating materials at the nanoscale (10^−9^) to create novel therapeutic modalities that exhibit unique properties distinct from their bulk counterparts [152]. At the nanoscale, various biological mechanisms in the human body come into play, which may allow nanoparticles and nanomaterials to potentially penetrate natural barriers and interact with DNA or small proteins at different levels. Fig. 3 shows an overview of different traits of nanoparticles. The surface-to-volume ratio is such that the surface properties become an intrinsic parameter of a particle or material's potential actions. Therefore, coating particles and functionalizing their surfaces is common to increase biocompatibility and ensure a highly selective binding to the desired target [153].

The field of nanomedicine has the potential to revolutionize modern medicine by enabling the development of precision medicine, personalized therapies, early disease detection, and targeted drug delivery [152]. Implementing nanomedicine has the potential to significantly enhance the efficacy of existing treatments while mitigating their side effects. As such, it represents a crucial field of research for the healthcare industry and is anticipated to profoundly impact the landscape of medicine.

Recently, renal nanomedicine has been rapidly expanding, utilizing nanoparticles as diagnostic and treatment tools with various imaging techniques. Numerous FDA-approved nanodrugs and nanoparticles are available for treating kidney diseases, including kidney cancer and chronic kidney disease. Some of these are even under clinical trials [154,155]. However, the treatment of kidney stones is an area that remains less explored and needs a nanodrug solution. Kidney stones are unique in size, hardness, chemical composition, and location within the urinary system. The current methods for breaking up kidney stones may only be effective in some cases. In a recent study, Williams et al. highlight the potential of nanomedicine in treating various kidney diseases, including hypertension, acute kidney injury, chronic kidney disease, glomerular disease, and kidney cancer. Further, it also highlights the absence of a nanodrug solution for kidney stones is a significant issue, as it affects a considerable portion of the population [147].

An innovative approach proposed by Vordos et al. involves using gold nanoparticles and a laser beam for kidney stone fragmentation, highlighting the potential of nanotechnology in addressing kidney stone-related issues [156]. They introduced the concept of a nanodrug utilizing gold nanoparticles with the potential for treating kidney stones. The design of nanoparticles to fit within stone pores and the specification of energy for laser-based fragmentation are guided by crucial parameters such as crystal thickness and pore diameter, which can be determined through techniques such as SAXS and nitrogen porosimetry, enabling the morphological characterization of calculi.

The treatment of urolithiasis could potentially benefit from the use of various nanoparticles, which should be the focus of future research efforts. For instance, the use of silver nanoparticles (AgNps) synthesized with seaweed extracts for anti-urolithiasis purposes is a promising avenue to explore. Targeted drug delivery of seaweed bioactive compounds using AgNps could be a cutting-edge method. It combines the natural therapeutic properties of seaweed bioactive compounds with the advanced delivery capabilities of nanoparticles to offer more effective and safer kidney stone treatments. AgNps have demonstrated several therapeutic properties, including the ability to prevent microbial growth, inhibit tumor growth, and act as antioxidants [157]. Therefore, they are a compelling candidate for further research in the context of kidney stone treatment. Although some studies have explored the anti-urolithiasis activity of AgNPs synthesized using herbal plant extracts. Recent research by Das et al. has found that *Phlogacanthus thyrsiformis* flower extract based AgNps can effectively hinder the development of struvite crystals *in vitro* [158]. In addition, the study examined male wizard rats with induced urolithiasis using the extract and nanoparticles. As a result, the rats’ weight increased, their water consumption normalized, and their urine volume and calcium, phosphorus, and magnesium balance were restored. The treatment also prevented crystal formation and kidney damage. The study identifies two possible mechanisms for inhibiting crystal growth: the formation of complexes between phytoconstituents and nanoparticles with Mg^2+^ ions, which limits their availability for crystal growth, and adsorption on the crystal surface, which hinders nucleation. Another study by the same group reported that *Oxalis corniculata* Linn. Leaves aqueous extract and its biofabricated AgNps exhibited potent antibacterial activity against gram-negative and gram-positive bacteria. The extract and nanoparticles also revealed the ability to inhibit and dissolve struvite kidney stones, suggesting the potential for reducing the risk of urinary tract infections and urolithiasis [159]. Another study evaluated the efficacy of *Tragia involucrata* extract and its biofabricated AgNPs in preventing urolithiasis [160]. Both the extract and AgNps demonstrated effective inhibition of struvite crystal growth, with AgNps showing greater inhibition. Rats with CaOx urolithiasis induced by ethylene glycol experienced weight loss, abnormal urine composition, increased water intake, and elevated serum and kidney parameters. However, the administration of the extract and AgNps protected the kidney tissues, which was confirmed through histopathological analysis. *T. involucrata* and its AgNps may have anti-urolithiatic properties due to the presence of bioactive compounds, the formation of stable AgNps with crystal growth inhibitory properties, improved crystal solubility, and restoration of urinary parameters. These properties prevent supersaturation and subsequent crystal formation. In a similar study, the potential of AgNPs synthesized using *Bryophyllum pinnatum* was tested for reducing crystal deposition and protecting against kidney injury in an ethylene glycol-induced urolithiasis rat model [161]. The results showed that the AgNPs were able to significantly reduce crystal deposition, restore renal function markers, and improve histopathology in kidney tissues. These findings suggest that *B. pinnatum*-mediated AgNPs have the ability to effectively reduce urolithiasis and protect against kidney injury, likely due to the presence of bioactive substances such as quercetin and kaempferol, which possess antioxidant properties. Another study investigated the eco-friendly utilization of extracts from *Annona muricata* and *Trigonella foenumgraceum* leaves to create silver nanoparticles [162]. The nanoparticles were then tested for their antiurolithiatic properties by examining their effect on CaOx crystallization in synthetic urine. The extracts' phenols, saponins, and flavonoids aided in the bioreduction of Ag^+^ to Ag0 during AgNP synthesis. Moreover, polyphenolic compounds like quercetin had a direct impact on calcium oxalate crystallization.

In a study by Bi et al., it was discovered that combining curcumin-loaded gold nanoparticles and *Pluchea indica* root extract effectively decreased stone formation in male Wistar rats. This was achieved through the reduction of oxidative stress and inhibition of kidney crystallization by utilizing their anti-inflammatory and antioxidant characteristics [163]. Similarly, horse gram flour extracts and their biosynthesized AgNPs demonstrated anti-urolithiatic and anti-diabetic properties by inhibiting calcium oxalate crystallization, increasing glucose uptake, and reducing oxidative stress [164]. Although plant extracts have been widely studied for nanoparticle synthesis, seaweed-based nanoparticles have also shown promising results in addressing urolithiasis. Seaweed nanoparticles offer multiple advantages, including their abundance, sustainability, and eco-friendly nature. However, the exact mechanism of action for treating complex conditions like urolithiasis is not yet fully understood. Seaweeds, with their unique phytochemicals such as polysaccharides and bioactive compounds, may enhance the therapeutic properties of the biosynthesized nanoparticles. Furthermore, seaweed extracts are recognized for their biocompatibility, safety, and potent antimicrobial properties, making them suitable for clinical use. Additionally, the specific bioactive properties derived from the unique marine environment position seaweed-based nanoparticles as a promising option for innovative and effective therapeutic applications. Therefore, future focus should also prioritize the exploration of seaweed-based nanoparticles, which have shown more extensive and promising results.

### 4.1 Seaweed-based AgNps synthesis

**Fig. 4:**
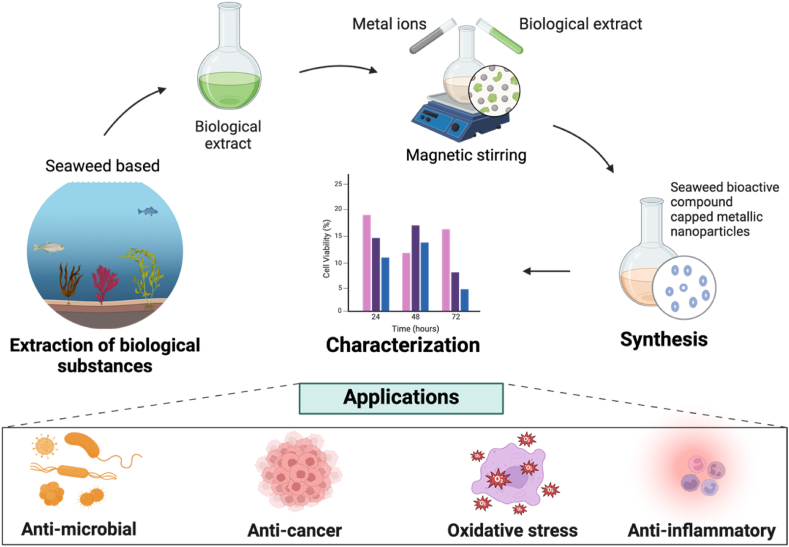
Seaweed-based AgNps synthesis and its therapeutic applications (Created with BioRender.com).

The synthesis of metallic nanoparticles using seaweed extracts is a method that is both environmentally friendly and less hazardous to biological systems. Seaweed has the remarkable ability to accumulate metals and reduce metal ions quickly. During the process of synthesizing metallic nanoparticles, biomolecules found in seaweed act as both reducing and capping agents (Fig. 4). These nanoparticles have potent therapeutic properties and serve as a promising alternative to plant-based nanoparticles [165]. Among the various types of seaweed-based metallic nanoparticles, AgNps hold particular appeal due to their unique optical, physical, and biological characteristics, as well as their exceptional electrical conductivity. Their medicinal properties span a wide range, including anticancer, antimicrobial, anti-inflammatory, and antioxidant activities.

AgNps are known for their high antimicrobial activity due to their small size and large surface area, which allows them to interact with microbial cells, penetrate them easily, and alter their membrane structure, ultimately leading to cell death. Recent studies have identified extracts of *Sargassum cinereum* (Phaeophyceae) [166] and *E. cava* (Phaeophyceae) [167] as sources for AgNps synthesis have strong antimicrobial properties. Additionally, AgNps derived from seaweeds like *Enteromorpha compressa* (Chlorophyta) [168] and *U. lactuca* (Chlorophyta) [169] have been found to be effective anticancer agents.

The antioxidant and free radical scavenging properties of AgNps are dependent on the bioactive compounds present in seaweeds, and their effectiveness can be enhanced by increasing the concentration of AgNps. Studies have demonstrated the antioxidant activity of AgNps synthesized by seaweeds such as *E. cava* (Phaeophyceae) [167], *Desmarestia Antarctica* (Phaeophyceae), and *Iridaea cordata* (Rhodophyta) [168]. Exhaustive reviews are available on the antimicrobial, anticancer, and antioxidant activities of AgNPs [[169], [170], [171]].

While several reports detail the therapeutic properties of seaweed-mediated nanoparticles in treating various kidney-related diseases [172,173], no attempts have been made to use seaweed-based nanomedicine to treat urinary calculi. Therefore, exploring the potential of seaweed-based nanomedicine for urolithiasis represents a new and exciting area of research.

### 4.2 Mechanism of action

**Fig. 5:**
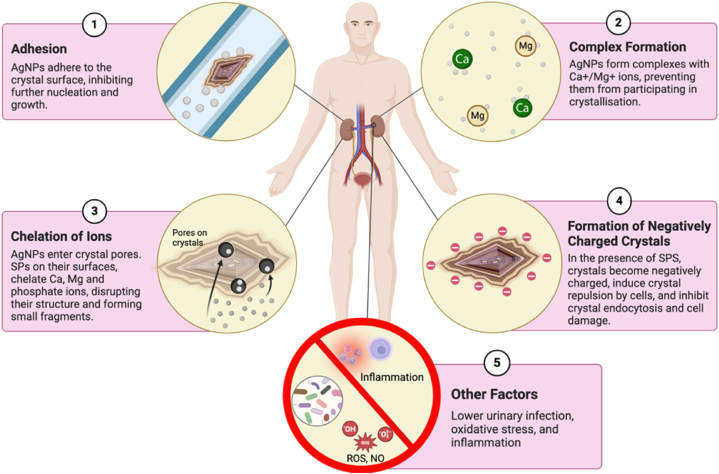
The potential antiurolithiatic effects of seaweed-derived bioactive compounds and their biofabricated AgNps (Created with BioRender.com).

The proposed mechanisms for the antiurolithiatic effects of seaweed-derived bioactive chemicals and their biofabricatedAgNps suggest a comprehensive approach to preventing kidney stone formation and recurrence (Fig. 5). AgNPs can adhere to the surface of crystals and effectively inhibit any subsequent nucleation and growth of these crystals, forming a barrier against the development of kidney stones. By inhibiting crystal expansion, AgNps play an essential role in minimizing the buildup of kidney stones. Studies have shown that bioactive compounds present in seaweed, such as SPS and phenolic compounds, can interact with crystal surfaces, reducing or preventing the formation of kidney stones. These chemicals can prevent stone formation by interrupting the early phases of crystal formation. Seaweed-mediated AgNps can form complexes with calcium (Ca+) and magnesium (Mg+) ions, acting as protective shields to prevent these ions from participating in crystallization. Ca+ and Mg + ions play an essential part in producing various kidney stones; therefore, removing them from the equation slows crystal growth and reduces the risk of stone formation. AgNPs are well-suited for entering the pores of kidney stones because of their small size and unique characteristics. The surfaces of AgNPs contain SPS rich in anionic groups, which can chelate calcium, magnesium, and phosphate ions in kidney stones, disrupting their crystalline structure and causing them to break apart [174]. This fragmentation process effectively reduces large kidney stones to smaller ones, which can be easily discharged from the urinary canal. Additionally, in the presence of SPS, the crystals become negatively charged and are repelled by the negatively charged cell membrane, inhibiting crystal endocytosis and subsequent cell damage. Additionally, by lowering urinary infection, oxidative stress, and inflammation, seaweed-based AgNPs may lessen the environment that favors the development and growth of kidney stones.

The potential of kidney stone prevention and treatment with seaweed-based AgNPs is promising. These substances not only halt the formation of new crystals but also break down existing stones, prevent infection, and reduce inflammation. This comprehensive approach could significantly reduce the development and recurrence of kidney stones, offering a potential breakthrough in urology and nephrology.

While seaweed-based nanoparticles show great promise in urology and kidney stone management, further *in vitro* investigations and clinical trials are imperative to ensure their safety and effectiveness for human use. Rigorous scientific testing is necessary to validate these approaches and establish their viability for human application, underscoring the need for continued research in this area.

## 5 Conclusion

Urolithiasis has been the subject of extensive research, yet a comprehensive understanding of its pathological mechanisms and effective drug development remains elusive. The involvement of multiple mechanisms in the pathogenesis of urolithiasis presents a significant challenge in the development of antiurolithiatic drugs. Recurrence of stones is a major issue in treating the disease, and further studies are needed to unravel the molecular mechanisms involved in urolithiasis and develop drugs that can address multiple targets. Future prospective studies should prioritize the prevention of stone recurrence, as a higher proportion of patients experience recurrence than those who suffer from a single onset of the disorder.

Seaweeds are a rich source of numerous metabolites with biological properties, including polysaccharides, proteins, lipids, carotenoids, vitamins, sterols, enzymes, minerals, antibiotics, dietary fiber, essential fatty acids, and various other components. Research on seaweeds has demonstrated their diuretic, antioxidant, anti-inflammatory, analgesic, antimicrobial, anti-apoptotic, enzyme inhibitory, and antiurolithiatic properties. Given that urolithiasis is a multifactorial disease, seaweed with multiple therapeutic properties may be a safe and effective drug for treating renal calculus. However, the active ingredients present in seaweeds and the seaweed-based nanoparticles that can dissolve kidney stones or reduce the chance of recurrence require further investigation.

Prospective studies that address medication surveillance of allopathy drugs administered in urolithiasis are crucial to better understanding alternative treatment procedures involving natural products that could help prevent a relatively more significant proportion of patients from suffering from side effects. The safety, therapeutic targeting, and bioavailability of seaweed metabolites can be improved by nanoparticles. Additional studies focusing on the isolation and determination of the efficacy and safety of active compounds in seaweeds and their bio-fabricated nanoparticles that could ameliorate urolithiasis would open a new avenue for treating urolithiasis.

## CRediT authorship contribution statement

**Dhanya Raj C. T:** Writing – review & editing, Writing – original draft, Formal analysis, Conceptualization. **Vivekanandan Palaninathan:** Writing – review & editing, Writing – original draft, Validation, Software, Conceptualization. **Surabhi Kandaswamy:** Writing – review & editing, Validation. **Vimal Kumar:** Writing – review & editing. **Rathinam Arthur James:** Writing – review & editing, Validation, Supervision, Resources, Formal analysis, Conceptualization.

## Data availability statement

No data was used for the research described in the article.

## Consent for publication

Not applicable.

## Declaration of competing interest

“The authors declare no competing interests.”

## Abbreviations

CaOx -: Calcium oxalate
CaP -: Calcium phosphate
ROS -: Reactive oxygen species
NADPH oxidase -: Nicotinamide adenine dinucleotide phosphate oxidase
RAS -: Rennin angiotensin system
IL-1β -: Interleukin-1β
NLRP3 -: NOD-LRR-and pyrin domain-containing protein3
TLRs: Toll-like receptors
MAP -: Magnesium ammonium phosphate
CO_2_ -: Carbon dioxide
SPs -: Sulfated polysaccharides
PLA_2_ -: Phospholipase A2
p38 MAPK: p38 mitogen-activated protein kinase
PI3K/Akt: Phosphoinositide 3-kinase
UTI -: Urinary tract infection
COD -: Calcium oxalate dehydrates
COM -: Calcium oxalate monohydrate
AgNps -: Silver nanoparticles
